# Mutant MMP-9 and HGF Gene Transfer Enhance Resolution of CCl_4_-Induced Liver Fibrosis in Rats: Role of ASH1 and EZH2 Methyltransferases Repression

**DOI:** 10.1371/journal.pone.0112384

**Published:** 2014-11-07

**Authors:** Hussein Atta, Mahmoud El-Rehany, Olfat Hammam, Hend Abdel-Ghany, Maggie Ramzy, Martin Roderfeld, Elke Roeb, Ayman Al-Hendy, Salama Abdel Raheim, Hatem Allam, Heba Marey

**Affiliations:** 1 Department of Surgery, Faculty of Medicine, Minia University, El-Minia, Egypt; 2 Department of Biochemistry, Faculty of Medicine, Minia University, El-Minia, Egypt; 3 Department of Pathology, Theodor Bilharz Research Institute, Giza, Egypt; 4 Department of Gastroenterology, Justus-Liebig University Giessen, Germany; 5 Department of Obstetrics and Gynecology, Georgia Regents University, Augusta, Georgia, United States of America; University of Navarra School of Medicine and Center for Applied Medical Research (CIMA), Spain

## Abstract

Hepatocyte growth factor (HGF) gene transfer inhibits liver fibrosis by regulating aberrant cellular functions, while mutant matrix metalloproteinase-9 (mMMP-9) enhances matrix degradation by neutralizing the elevated tissue inhibitor of metalloproteinase-1 (TIMP-1). It was shown that ASH1 and EZH2 methyltransferases are involved in development of liver fibrosis; however, their role in the resolution phase of liver fibrosis has not been investigated. This study evaluated the role of ASH1 and EZH2 in two mechanistically different therapeutic modalities, HGF and mMMP-9 gene transfer in CCl_4_ induced rat liver fibrosis. Liver fibrosis was induced in rats with twice a week intraperitoneal injection of CCl_4_ for 8 weeks. Adenovirus vectors encoding mMMP-9 or HGF genes were injected through tail vein at weeks six and seven and were sacrificed one week after the second injection. A healthy animal group was likewise injected with saline to serve as a negative control. Rats treated with mMMP-9 showed significantly lower fibrosis score, less Sirius red stained collagen area, reduced hydroxyproline and ALT concentration, decreased transforming growth factor beta 1 (TGF-β1) mRNA and lower labeling indices of α smooth muscle actin (α-SMA) and proliferating cell nuclear antigen (PCNA) stained cells compared with HGF- or saline-treated rats. Furthermore, TIMP-1 protein expression in mMMP-9 group was markedly reduced compared with all fibrotic groups. ASH1 and EZH2 protein expression was significantly elevated in fibrotic liver and significantly decreased in mMMP-9- and HGF-treated compared to saline-treated fibrotic livers with further reduction in the mMMP-9 group. Conclusion: Gene transfer of mMMP-9 and HGF reduced liver fibrosis in rats. ASH1 and EZH2 methyltransferases are significantly reduced in mMMP-9 and HGF treated rats which underlines the central role of these enzymes during fibrogenesis. Future studies should evaluate the role of selective pharmacologic inhibitors of ASH1 and EZH2 in resolution of liver fibrosis.

## Introduction

Liver fibrosis and its end-stage sequela of cirrhosis are major causes of morbidity and mortality worldwide and result from different etiologies of chronic liver injury. The high morbidity and mortality associated with fibrosis/cirrhosis underscores the need for novel preventive and therapeutic approaches [1]. Fibrosis accumulation is a dynamic process resulting from a wound-healing response involving pathways of fibrogenesis and inflammation [2]. Fibrosis reflects the imbalance between matrix production and degradation [3]. During liver injury, hepatocyte necrosis and apoptosis instigate inflammatory signaling by chemokines and cytokines resulting in recruitment of immune cell populations, and activation of fibrogenic cells, culminating in the deposition of extracellular matrix (ECM) [4]. However, a major determinant of progressive fibrosis is failure to degrade the increased interstitial matrix [3].

ECM degradation and remodeling is controlled by a fine balance between matrix metalloproteinases (MMPs) and tissue inhibitors of matrix metalloproteinases (TIMPs). TIMP-1, the most important endogenous inhibitor of most MMPs, plays a crucial role in the pathogenesis of liver fibrosis and represents an important therapeutic target in the design of antifibrotic strategies for chronic liver disease [5], [6]. Previous reports demonstrated that TIMP-1 attenuates spontaneous resolution of liver fibrosis by the combination of a net reduction of the MMP activity and suppression of apoptosis in activated hepatic stellate cells (HSCs) [7], [8]. The increased proteolytic activity in fibrotic liver is counteracted by elevated TIMP-1 activity. Specific neutralization of TIMP-1 with catalytically inactive MMP-9 was shown to inhibit hepatic fibrogenesis in mice [6]. MMP-9 was chosen for construction of TIMP-1 antagonists due its high affinity (K_i_ values 50 pM) to TIMP-1 [9], [10]. The catalytically inactive MMP-9 was preferentially used over the wild-type enzyme because the enzyme activity of the wild-type MMP-9 is known to play a role in tumor cell invasion and deemed unsuitable for antifibrotic therapy. The enzymatically inactive MMP-9 was constructed by substitution of glutamic acid at position 402, which is essential for the catalytic mechanism, with glutamine resulting in mutant MMP-9 (mMMP-9) E402Q. The single substitution of the negatively charged to a neutral amino acid abolished the enzymatic activity but did not alter the 3-dimensional structure, as evidenced by its effectiveness in binding and antagonizing TIMP-1 [6], [11].

Hepatocyte growth factor (HGF) is a potent mitogen for a variety of cells including mature hepatocytes [12]. HGF promotes liver regeneration both in normal [13] and diseased liver [14]. HGF has been used effectively to enhance resolution of experimental liver fibrosis/cirrhosis [15]–[17] and regeneration following resection of fibrotic liver [18]. The enhanced regeneration potential mediated by HGF has been attributed to multiple biological effects. HGF exerts a protective effect against hepatocyte injury [19], [20] and apoptosis [21] through upregulation of the antiapoptotic protein Bcl-xL [22], [23]. In rats with liver cirrhosis, HGF suppressed the increase of transforming growth factor-β1 (TGF-β1), which plays an essential role in the progression of liver cirrhosis, decreased profibrogenic markers as collagen expression and α-SMA [17] and inhibited fibrogenesis [24]. HGF has also been shown to prevent tissue fibrosis in the kidneys by increasing MMP-9 and suppressing expression of TIMP-1 [25].

ASH1 (absent, small, or homeotic disc 1) is a member of trithorax group of proteins involved in maintaining the active state of gene expression. ASH1 is a histone methyltransferase that targets lysine 4 on histone H3 [26]. Trimethylation of K4 on H3 by ASH1 leads to gene activation [27]. It has recently been shown that ASH1 is a positive regulator of multiple profibrogenic genes. In activated HSCs, ASH1 was found to directly bind to TIMP-1, α-SMA, collagen I, and TGF-β1 gene promoters and increases levels of associated histone H3 lysine 4 trimethylation resulting in their activation. The activation of these genes is necessary for the onset and progression of fibrogenesis [28]. In HSCs, ASH1 expression is regulated both at the transcriptional and translation levels. Thus, although quiescent HSCs (qHSCs) contain some ASH1 transcript which increases with HSC activation, however, no detectable protein was found in activated HSCs [28].

EZH2, a histone H3 lysine-27-specific methyltransferase, is the catalytic subunit of the multi-enzyme complex polycomb repressive complex 2 (PRC2) and is involved in chromatin compaction and gene repression [29]. EZH2 regulates cell proliferation and differentiation by silencing polycomb group target genes, and has been linked to the aggressiveness of human cancers, including hepatocellular carcinoma [30]. Moreover, EZH2 promotes epithelial–mesenchymal transition, a process that is associated with both liver carcinogenesis and injury and repair process [31]. Recently, the role of EZH2 in the development of liver fibrosis has been investigated. EZH2 mRNA and protein expression were found to be absent in qHSCs and were induced with activation in response to CCl_4_ injury [32]. It was shown that stimulation of EZH2 expression during myofibroblast transdifferentiation and subsequent methylation of H3K27 to form a repressive chromatin structure in the 3′ exons of peroxisome proliferator-activated receptor-gamma (PPARγ) gene results in downregulation PPARγ expression [32]. It is well known that transcriptional silencing of the PPARγ gene is required for conversion of qHSCs into myofibroblasts. This type of gene silencing mechanism that occurs at PPARγ genes in activated HSCs is required for these cells to acquire their inflammatory and fibrogenic phenotype [33].

The primary aim of this study was to examine if there is a synergistic antifibrotic effect of a combination of two mechanistically different therapeutic modalities, degradation of ECM with mMMP-9 (E402Q) neutralization of TIMP-1 and freeing of the endogenous catalytically active MMP-9 and hepatocyte protection with the multifunctional growth factor HGF, on CCl_4_-induced liver fibrosis in rats during active fibrogenesis. Additionally, we investigated the role of ASH1 and EZH2 methyltransferases in mMMP-9 and HGF gene therapy-induced resolution of CCl_4_-model of rat liver fibrosis.

## Material and Methods

### Ethics statement

All animals received humane care in accordance with the National Institutes of Health Guide for the Care and Use of Laboratory Animals. The study protocol was approved by the Faculty of Medicine Minia University Council.

### Vector Construction and CCl_4_ Liver Fibrosis Model

The E1-deleted replication-deficient recombinant adenovirus type 5 vector expressing human hepatocyte growth factor gene (HGF) or mutant mouse MMP-9 (mMMP-9) under the transcriptional control of the cytomegalovirus (CMV5) immediate-early promoter/enhancer was constructed using standard techniques as we have described previously [34]. To test the study aims we used the validated and reproducible rat model of chronic CCl_4_ liver injury [35]. CCl_4_ was administered for 8 weeks to induce advanced fibrosis/cirrhosis and was continued during gene transfer and until sacrifice in order to mimic clinical scenario where the fibrosis inducing agent continues to be operative during antifibrosis treatment. Experiments that discontinue the fibrosis inducing agent before administering therapeutic treatments may be confounded by the possibility of spontaneous resolution of fibrosis that occur due to cessation of liver injury [5]. We used sufficient numbers of animals per group to overcome individual heterogeneity in fibrosis progression [36]. In order to limit the vector-associated inflammatory response and toxicity, we used an adenovirus vector dose that is at the lower range of the reported HGF adenovirus doses used in similar experiments [16], [18], [37] but lower than the only published mMMP-9 adenovirus study [6]. We have confirmed transduction of the cirrhotic livers with the detection of the adenovirus E4 gene product by PCR analysis. Furthermore, in order to augment gene transfer we administered two doses of the therapeutic gene one week apart.

### Experimental Procedure

Fibrosis was induced in adult male Wistar rats by twice a week intraperitoneal injection of carbon tetrachloride (CCl_4_, 0.8 mL/kg as a 1∶1 mixture with mineral oil) for 8 weeks [6]. At weeks 6 and 7 adenoviral vector (1.0×10^8^ pfu each) encoding human hepatocyte growth factor (HGF) alone, mutant (E402Q) matrix metalloproteinase-9 (mMMP-9) alone or both, or saline were injected through tail vein. Healthy animals were injected with saline twice a week for 8 weeks and served as control. Animals were sacrificed at the end of week eight. Rats were anesthetized with intramuscular injection of 60 mg/kg ketamine hydrochloride (Sigma-Tec, Egypt) before sacrifice via cardiac puncture exsanguination. Liver tissue samples from the right median lobe were snap-frozen in liquid nitrogen or fixed in 10% buffered formalin.

### Expression Analyses

Total RNA was purified from homogenized liver specimen using RiboZol RNA Extraction reagent (Amresco, Solon, USA) following the manufacturer's instructions. Isolated total RNA (5 µg) was used as template to generate cDNA using OneStep RT-PCR Kit (Qiagen, UK). PCR amplification was performed in a DNA thermal cycler (Progene; Techne Ltd., Duxford, United Kingdom). For PCR E4 gene detection, genomic DNA was isolated using a QIAamp Tissue Kit (Qiagen Inc., Valencia, CA, USA) [13]. Nucleic acid concentrations were determined by spectrophotometer (Genova Plus, Bibby Scientific, UK). Primers and amplicon size are listed in Table S1 in File S1. The intensity of the PCR product bands were quantified using gel documentation system software (Biometra GmbH, Germany).

### Enzyme-Linked Immunosorbent Assay

Liver homogenates were used for the determination of total protein concentration by the Coomassie binding method [38]. Enzyme-linked immunosorbent assay (ELISA) kits were used to measure the concentration of rat MMP-9, human HGF, rat TIMP-1 using Quantikine ELISA kit (R & D Systems Inc., Minneapolis, USA), and rat hydroxyproline ELISA kit (Sunred Biological Technology, Shanghai, China) according to the manufacturers' instructions. Serum alanine aminotransferase (ALT) concentration was measured by chemical analyzer (TC 3300-Teco diagnostic, Anaheim, USA) using kinetic kits (HUMAN Biochemical and Diagnostic, Wiesbaden, Germany) according to the standard procedures.

### Slot Blot Assay

A total of 50 µg proteins were transferred onto pre-wetted nitrocellulose membranes (Whatman, Maldstone, UK) in TBST (10 mM Tris, 0.15 M NaCl, 0.05% Tween-20, pH 8.0) under vacuum using a Slot Blot Filtration Manifold (Hoefer Inc., MA, USA) according to the manufacturer's instructions. The membranes were removed and incubated in TBST containing bovine albumin for 30 min. The membranes were incubated for 1 h at room temperature with mouse monoclonal anti-ASH1 (ab 50981, Abcam, Cambridge, UK) and mouse monoclonal anti-EZH2 (AC22; #3147, Cell Signaling Technology, Danvers, MA, USA) primary antibodies diluted 1: 1000 in TBST. EZH2 mouse mAb detects endogenous levels of total EZH2 protein. The membranes were then washed in TBST three times, 10 min each and incubated with goat anti-mouse IgG alkaline phosphatase conjugated secondary antibody (Sigma-Aldrish, Inc., USA) diluted 1∶7500 in TBST for 30 min. The membranes were washed in TBST three times for 15 min each. After this, the membranes were incubated in freshly prepared BCIP-NBT color development solution (Sigma-Aldrish, Inc., USA) for 10 to 30 min. Then the stopper solution (20 mM Tris-HCl and 5 mM EDTA, pH 8) was added to the membranes at room temperature with gentle rocking. The membranes with developed bands were dried and photographed. Quantification of band intensity was performed using the Image J software (NIH, Bethesda, MD, USA).

### Liver Histology and Morphometric Analysis

Quantitative analysis of collagen in Sirius Red-stained liver sections was performed using imaging analysis software (Axiovision L.E. 4.8; Carl Zeiss MicroImaging, GmbH, Germany). Briefly, paraffin sections of 20 µm thickness were stained in 0.1% Sirius red F3B (SR) in saturated picric acid [6]. The red-stained area (µm^2^) was measured in five consecutive fields (×50). Fibrotic area percent was calculated relative to the total area examined. Morphometric analysis of hepatic fibrosis score was performed on Hematoxylin and Eosin (H&E) and Masson's trichrome (MT) stained liver sections (X400) using semiquantitative fibrosis scores as described previously [39], [40]. Healthy liver was classified as S0 stage, no fibrosis. S1 stage, expansion of fibrosis in portal area, localized perisinusoidal and intralobular fibrosis; S2 stage, peripheral fibrosis in portal area, formation of fibrous septum, retention of intralobular architecture; stage S3, fibrous septum accompanied by intralobular structural disorders, no hepatic cirrhosis; S4 stage, early hepatic cirrhosis characterized by bridging fibrosis with pseudo-lobule formation.

### Liver Immunohistochemistry

Paraffin sections were deparaffinized and rehydrated. Endogenous peroxidase was inactivated by incubation in 0.3% hydrogen peroxide in absolute methanol for 30 min. Sections were incubated in 5% skim milk for 30 min at room temperature. Antigen retrieval was performed by microwave (700 W) treatment in 10 mM citrate buffer (pH 6.0) for 15 min. Sections were incubated overnight at 4°C with anti-rat proliferating cell nuclear antigen (PCNA) and α-smooth muscle actin (α-SMA) primary antibodies (Abcam, Cambridge, USA) at dilution of 1∶100 and 1∶150, respectively. After washing with PBS, sections were incubated at room temperature for 30 min in secondary antibodies (Abcam, Cambridge, USA). A brown color was developed with 3 diaminobenzidine for 2–4 min, washed in distilled water and counterstained with Mayer's hematoxylin for 1 min at room temperature. The percent of positively stained brown nuclei (PCNA) or brown cytoplasm (ASMA) in 10 successive fields (X400) were calculated [13].

### Statistical Analysis

Results are expressed as mean ± standard deviation (SD) or median (25th–75th interquartile range, IQR) for normally or not-normally distributed groups, respectively. Kolmogorov-Smirnov test was applied to test for a normal distribution. Differences among normally distributed groups with continuous data were compared using one-way ANOVA and Scheffe post-hoc multiple comparisons test, while Kruskal-Wallis nonparametric test was used in not-normally distributed groups. Differences between groups in fibrosis score, a categorical variable, were detected using chi-square test. For all statistical analyses, 2-tailed tests were used. Statistical analysis was performed using the software Statistical Package for Social Sciences, SPSS version 13 (SPSS, Chicago, IL, USA). A *p* value of <0.05 was considered statistically significant.

## Results

### HGF and mMMP-9 Gene Transfer to the Fibrotic Liver

Transduction of the adenovirus vectors encoding HGF or mMMP-9 was confirmed by PCR detection of the essential E4 adenovirus gene expression in the liver tissues of transfected groups (Figure S1 in File S1).

**Figure 1:**
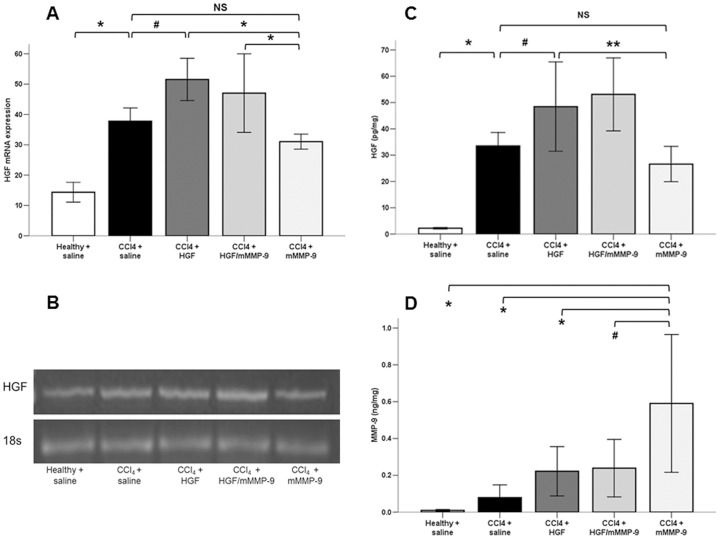
Analysis of HGF and MMP-9 gene expression. (A, B) RT-PCR gel of HGF mRNA expression. HGF mRNA expression was significantly (*, p<0.001) elevated in the saline-treated fibrotic (n = 14) compared to saline-treated healthy livers (n = 10) and further elevated (#, p<0.01) in the HGF-treated fibrotic (n = 13) compared with the saline-treated fibrotic and mMM-9-treated fibrotic (n = 10) livers (*, p<0.01). (C) Analysis of HGF protein concentration in the liver tissues. HGF protein concentration was significantly (*, p<0.001) elevated in the liver of the saline-treated fibrotic livers (n = 11) compared to saline-treated healthy livers (n = 10) and more elevated in the HGF-treated fibrotic (n = 14) compared to the saline-treated fibrotic (#, p<0.05) and mMMP-9-treated fibrotic (n = 10) livers (**, p<0.01). (D) Analysis of MMP-9 protein concentration in the liver tissues. MMP-9 protein concentration was significantly elevated in the mMMP-9-treated (n = 10) compared with healthy (n = 9), saline-treated fibrotic (n = 13) livers, HGF-treated (n = 12) livers (*, p<0.01) and HGF/mMMP-9-treated (n = 6) livers (#, p<0.05).

HGF mRNA expression and protein concentration (15.2 folds) were significantly elevated in the untreated fibrotic livers compared to healthy livers and were further elevated following treatment with HGF vector. In contrast, HGF mRNA and protein expressions in the livers of mMMP-9-treated group were no different than those in untreated fibrotic liver but were significantly lower than those of HGF-treated livers (Figures 1. A–C). HGF expressions in the livers of the combined HGF/mMMP-9-treated group were statistically not different from those of the HGF-treated group.

As expected, MMP-9 protein concentration was significantly elevated in the mMMP-9-treated livers compared with Healthy livers (97.5 folds), untreated fibrotic livers (9.1 folds) and HGF-treated fibrotic livers (3.6 folds). MMP-9 protein concentrations was elevated (10.6 folds) in the untreated fibrotic livers compared to healthy livers, in agreement with previous reports [8], [41], [42]. There was no difference in the MMP-9 protein concentrations in the livers of the HGF-treated or combined HGF/mMMP-9-treated livers compared with the untreated fibrotic livers (Figure 1.D).

### Mutant MMP-9 Reduces Fibrotic Markers and Improves Liver Function

We examined the effect of mMMP-9 and HGF gene transfer to the fibrotic liver on the expression of fibrotic markers and on serum ALT concentration. We found that the expression of all analyzed molecular markers of liver fibrogenesis was significantly elevated in liver tissues of the untreated fibrotic rats compared with those of healthy rats with TIMP-1 protein concentration elevated 252 folds.

**Figure 2:**
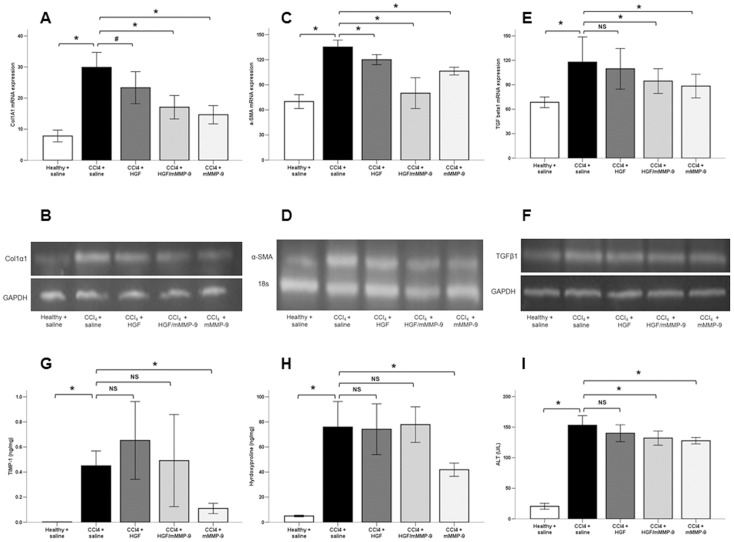
Effect of gene therapy on molecular markers of liver fibrosis. (A, B) Col1α1 mRNA expression was significantly elevated (*, p<0.001) in saline-treated livers compared with healthy livers (n = 10), and was significantly reduced with HGF (n = 14, #, p<0.05), mMMP-9 (n = 10, *, p<0.001) and HGF/mMMP-9 (n = 8, p<0.001) treatments compared with saline-treated fibrotic livers (n = 13). (C, D) α-SMA mRNA expression was significantly reduced with HGF (n = 14, p<0.001), mMMP-9 (n = 10, p<0.001) and HGF/mMMP-9 (n = 9, p<0.001) treatments compared with saline-treated fibrotic livers and was significantly elevated (p<0.001) in saline-treated fibrotic livers compared with healthy livers (n = 10). (E, F) TGF-β1 mRNA expression was significantly reduced with mMMP-9 (n = 10, p<0.01) and HGF/mMMP-9 (n = 9, p<0.01) treatment but not with HGF (n = 13, p = 0.87) compared with saline-treated fibrotic livers (n = 13). (G) TIMP-1 protein concentration was elevated (p<0.01) in saline-treated fibrotic livers (n = 9) and was further elevated in HGF-treated livers (n = 13) compared with healthy liver (n = 9). TIMP-1 concentration in fibrotic livers was significantly (p<0.001) decreased with mMMP-9 (n = 10) but not with HGF (p = 0.09) or with HGF/mMMP-9 (n = 9, p = 0.69) treatment. (H) Hydroxyproline concentration in saline-treated fibrotic livers (n = 10) was significantly elevated (p<0.001) compared with healthy livers (n = 10) and was significantly (p<0.001) decreased only with mMMP-9 treatment (n = 10) but not with HGF (n = 11, p = 0.86) or with HGF/mMMP-9 (n = 7, p = 0.98) treatments. (I) ALT serum concentration in saline-treated fibrotic rats (n = 14) was significantly elevated (p<0.001) compared with healthy rats (n = 10) and was significantly decreased with mMMP-9 (n = 10, p<0.001) and combined HGF/mMMP-9 (n = 9, p<0.01) but not with HGF (n = 14, p = 0.15) treatment.

Fibrotic rats treated with two injections of mMMP-9 showed significant reduction in all profibrogenic markers including col1α1 mRNA, α-SMA mRNA, TGF-β1 mRNA, TIMP-1 concentration, hydroxyproline content in liver tissues and serum ALT concentration compared to saline-treated fibrotic rats. Fibrotic rats treated with two injections of HGF showed significant reduction in only two profibrogenic markers, Col1α1 mRNA and α-SMA mRNA and no significant changes in other markers when compared with saline-treated fibrotic rats (Figure 2). This suggests that mMMP-9 gene transfer is more effective in enhancing resolution of liver fibrosis, at least in early phase of resolution, compared with HGF gene transfer.

### Mutant MMP-9 but not HGF Reverses Fibrosis Score and Collagen Deposition

**Figure 3:**
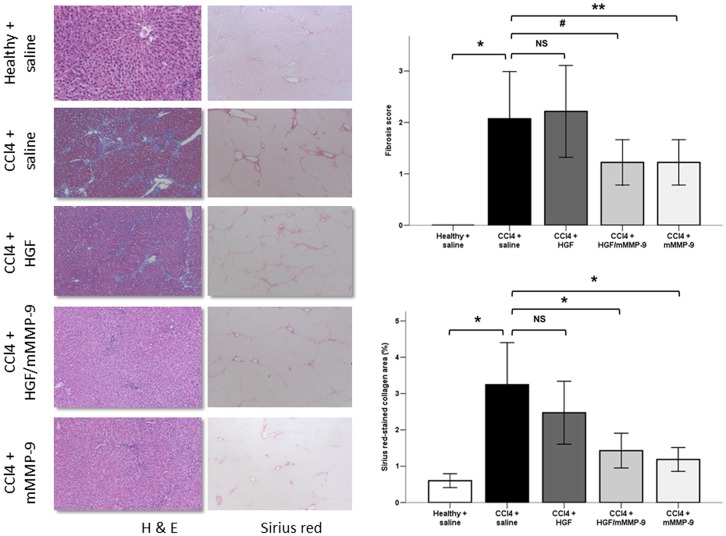
Histopathological findings in rat liver sections stained with H&E and Sirius-red. Fibrosis score was significantly elevated (*, p<0.001) in saline-treated fibrotic (n = 14) compared with healthy livers (n = 10). Fibrosis scores of HGF-treated livers (n = 14) were not statistical different from those of saline-treated livers (p = 0.71). However, fibrosis score was significantly reduced (**, p<0.01) in mMMP-9-treated (n = 9) and (#, p<0.05) in HGF/mMMP-9-treated (n = 9) compared to saline-treated fibrotic livers. (Original magnification ×200). Sirius red stained collagen area percent was significantly elevated (p<0.001) in saline-treated fibrotic livers (n = 14) compared with healthy livers (n = 10). Collagen area percent was significantly reduced in mMMP-9-treated (n = 9, p<0.001)) and in HGF/mMMP-9-treated (n = 9, p<0.01) but not in HGF-treated (n = 13, p = 0.19) compared to saline-treated fibrotic livers. (Original magnification, ×100).

To analyze histopathological changes in the livers following CCl_4_ treatment and gene transfer, we used H&E and MT staining to calculate fibrosis score and Sirius red staining to measure percent area of collagen deposition (Figure 3 and Figure S2 in File S1).

Gross and histopathological examination of livers of saline-treated healthy animals demonstrated normal appearing livers with normal histologic architecture and undetectable collagen deposition. Following twice weekly intraperitoneal injection of CCl_4_ for eight weeks, livers of saline-treated rats developed a median fibrosis grade score of 2.0 (interquartile range [IQR]: 2.0) indicating moderate fibrosis according to fibrosis grading system based on microscopic analysis of H&E and MT stained sections [39]. Treatment of fibrotic livers with two injections of vector encoding mMMP-9 gene or combined HGF/mMMP-9 genes resulted in significant reduction of liver fibrosis by 41 percent and a median fibrosis score grade of 1.0 (IQR: 1.0) indicating mild fibrosis. Unexpectedly, however, treatment of fibrotic liver with two injections of vector encoding HGF gene alone had no effect on the grade of liver fibrosis.

Collagen content of the liver was measured by quantitative analysis of collagen area percent in Sirius red-stained liver sections. Following twice weekly intraperitoneal injection of CCl_4_ for eight weeks, livers of saline-treated rats exhibited collagen area (M±SD) of 189233±67198 µm^2^ and percent of 3.2±1.15%. Treatment of fibrotic livers with two injections of vector encoding mMMP-9 gene or combined HGF/mMMP-9 genes resulted in significant reduction of liver collagen area (78090±41098 µm^2^ and 83469±28263 µm^2^) and percent by 60% and 55%, respectively. As with fibrosis score, treatment of fibrotic liver with two injections of vector encoding HGF gene alone showed non-significant reduction (157007±60688 µm^2^) in the collagen area of HGF-treated livers compared with saline-treated livers (Figure 3).

### Mutant MMP-9 Reduces α-SMA and PCNA Labeling Indices

**Figure 4:**
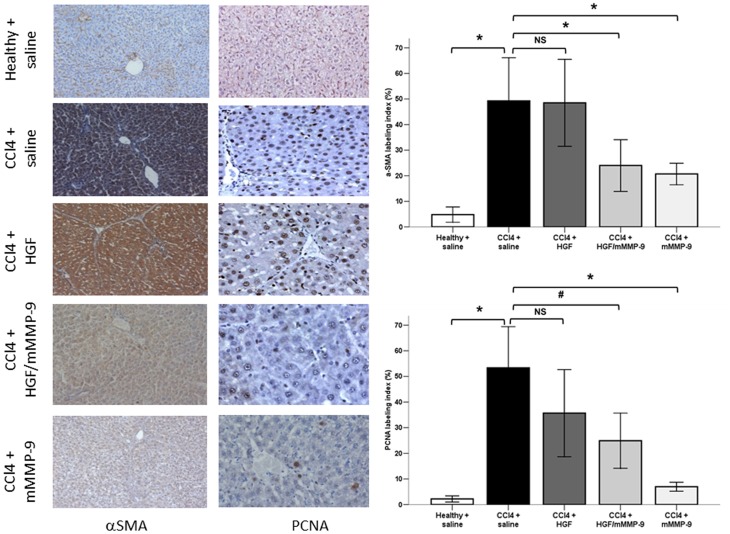
Immunohistochemical staining for α-SMA and PCNA in the liver. Bar graphs represent α-SMA and PCNA labeling indices (percent of positively stained brown cytoplasm or brown nuclei, respectively in 10 successive fields). Both α-SMA and PCNA indices were significantly elevated (*, p<0.001) in saline-treated fibrotic (n = 14 both) compared with healthy livers (n = 10 both) and both were significantly reduced (*, p<0.001) in mMMP-9-treated (n = 10 both) and in HGF/mMMP-9-treated (n = 9 both, *, p<0.001 and #, p<0.01, respectively) versus saline-treated fibrotic livers. Both α-SMA and PCNA labeling indices were not significantly different in HGF-treated fibrotic (n = 14, p = 0.99 and p = 0.06, respectively) from that in saline-treated fibrotic livers. (Original magnification ×400).

To document that resolution of liver fibrosis is associated with concomitant reduction of HSCs activation and global cellular proliferation we performed immunohistochemical analysis of α-SMA, a marker of HSC activation, and PCNA, a marker of cell proliferation in liver tissues. Our protocol of eight-week CCl_4_-induced fibrogenesis resulted in significant increase in α-SMA and PCNA labeling indices (12 folds and 24 folds, respectively) in livers of CCl_4_-treated compared with healthy rats. Although mMMP-9 treatment resulted in significant reduction of both α-SMA and PCNA labeling indices (58% and 87%, respectively) and to a lesser degree in combined treatment with HGF/mMMP-9 when compared with saline-treated fibrotic livers, however, treatment with HGF alone produced discordant results. While the effect of HGF gene treatment showed no reduction of α-SMA labeling index, it induced moderate significant reduction (33%) of PCNA labeling index when compared with saline-treated fibrotic groups (Figure 4).

### ASH1 and EZH2 Methyltransferases are Repressed during Fibrosis Resolution

**Figure 5:**
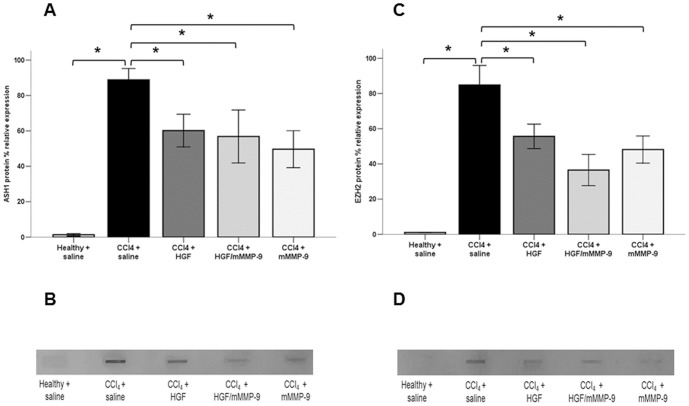
Analysis of ASH1 and EZH2 methyltransferases protein expression with Slot Blot. ASH1 (A, B) and EZH2 (C, D) protein expression were undetectable in the healthy livers. Both ASH1 and EZH2 protein expression markedly increased (p<0.01) following CCl_4_ treatment for eight weeks. Treatment with HGF, mMMP-9 or combined HGF/mMMP-9 resulted in significant (p<0.01) reduction of both ASH1 and EZH2 protein expression in fibrotic livers. There was no significant difference in the ASH1 or EZH2 protein expression between the HGF-, mMMP-9-, or HGF/mMMP-9-treated fibrotic livers. (n = 6 per group).

Expression of ASH1 and EZH2 histone lysine methyltransferases was measured using Slot Blot assay. We did not evaluate mRNA expression because previous report have demonstrated that although qHSCs contain some ASH1 transcript which increases with HSC activation, there was no detectable protein, suggesting that in addition to transcriptional control, ASH1 expression is at least in part regulated at translational or posttranslational levels [28]. Our analysis showed that ASH1 and EZH2 protein was minimally or not detectable in the liver of healthy saline-treated rats. Following eight weeks of CCl_4_ treatment ASH1 and EZH2 protein expression was markedly increased. Treatment of fibrotic livers with HGF or mMMP-9 gene transfer resulted in reduction of ASH1 (32 percent and 44 percent, respectively) and EZH2 (34 percent and 43 percent, respectively) proteins compared with saline-treated fibrotic livers. These results suggest that ASH1 and EZH2 histone lysine methyltransferases are repressed in the healthy liver. During fibrogenesis derepression of ASH1 and EZH2 resulted in their strong expression. Treatment with HGF or mMMP-9 induced repression of the activated ASH1 and EZH2 genes (Figure 5).

## Discussion

This study demonstrated several important findings. Firstly, mMMP-9 is more effective than HGF gene therapy in reducing CCl_4_-induced liver fibrosis in rats as supported by the documented reduction in all profibrogenic markers. Secondly, expression of HGF was increased following CCl_4_-induced fibrogenesis and was further elevated following HGF gene transfer. Moreover, most profibrogenic markers including TIMP-1, TGF-β1, α-SMA in addition to fibrosis score, collagen content, hydroxyproline concentration and serum ALT concentration were not reduced in livers treated with HGF gene vector. Thirdly, ASH1 and EZH2 lysine methyltransferases were markedly induced during progression of liver fibrogenesis but were repressed during the resolution phase of liver fibrosis following mMMP-9 or HGF gene transfer. However, ASH1 and EZH2 methyltransferases were more significantly repressed in livers of mMMP-9-treated rats than in livers of HGF-treated animals.

Both molecular analysis and histologic examination validated our experimental liver fibrogenesis protocol with CCl_4_ twice weekly intraperitoneal injection for eight weeks. Biochemical and molecular analyses showed consistent elevation of all profibrogenic markers, Col 1α1, TIMP-1, α-SMA, TGF-β1, hydroxyproline content and ALT concentration. Histologic examination demonstrated elevated fibrosis scores associated with advanced fibrosis and cirrhosis, while, Sirius red staining revealed increased collagen content in fibrotic livers. Immunohistochemical staining confirmed the expected elevation in α-SMA and PCNA labeling indices that accompany progressive fibrogenesis.

Here we used two mechanistically different strategies to inhibit experimental liver fibrosis. The first is HGF gene transfer which relies on its hepatocyte protective properties and the second is the newly developed MMP-9 mutant that effectively neutralizes TIMP-1, a crucial mediator of profibrogenic signals. We showed that the proteolytic inactive MMP-9 E402Q mutant inhibits CCl_4_-induced liver fibrosis in rats. Although in this study we employed a longer duration of CCl_4_ treatment and used a much smaller dose (<150 folds) of the adenovirus encoding mMMP-9, in order to limit the vector-associated inflammatory response than those reported originally by Roderfeld et al. [6], yet this dose was effective in transducing fibrotic livers and in significantly reducing all molecular and histologic parameters of liver fibrosis.

It has been shown that this catalytically inactive MMP-9 E402Q mutant binds TIMP-1 and neutralizes its effect in promoting liver fibrosis development [6]. Fibrotic livers exhibit an imbalance between MMPs and their inhibitors TIMPs. Specifically, TIMP-1 is considered a crucial factor in the pathogenesis of liver fibrosis [7], [43]. The antifibrotic mechanisms of mMMP-9 are the net combined effects of the matrix degradation by elevated endogenous MMP-9 as a result of scavenger of TIMP-1 and the effects of TIMP-1 reduction. Studies have shown that specific neutralization of TIMP-1 with antisense TIMP-1 plasmid or TIMP-1 antibody induces inhibition of HSC transdifferentiation to the active state and increase of apoptosis of activated HSCs and other fibrotic markers [6], [44], [45].

The effectiveness of HGF in inhibiting liver cirrhosis has been documented by a large body of literature [22], [24], [46], [47]. Here we found that HGF gene transfer to the fibrotic liver at the same dose as that used in mMMP-9 gene transfer did not inhibit all fibrosis markers as mMMP-9 did. Despite the fact that HGF treatment resulted in significant inhibition of key profibrogenic molecules such as Col1α1 and α-SMA mRNAs and PCNA labeling index, and upregulated PPARγ, an antifibrogenic transcription factor, HGF failed to reduce other important parameters of liver fibrosis including TGF-β1 mRNA, α-SMA labeling index, hydroxyproline content, serum ALT concentration, Sirius red-stained collagen area and fibrosis score. Although the design of our investigation does not allow us to make firm conclusion regarding the partial effectiveness of HGF in enhancing the resolution of liver fibrosis, however, we can propose the following explanations.

Firstly, we can speculate that the lower efficacy of the HGF in inhibiting liver cirrhosis is due to disruption of downstream HGF signaling pathway that conveys the hepatoprotective and antifibrotic effects. It has been shown previously that the intrahepatic expression of HGF specific receptor c-Met decreases at an early stage of cirrhosis development and significantly decreases at the time of cirrhosis manifestation [48] and that c-Met rather than HGF expression correlated more closely with proliferative index of liver lesion [49]. Thus the highly elevated HGF in the livers of HGF-treated rats in our study suggests diminished uptake of HGF by the decreased c-Met receptors in advanced fibrosis and cirrhosis that developed following 8 weeks of CCl_4_ administration. The direct consequence of decreased c-Met receptors is suppression of the HGF/c-Met signaling pathway and diminished antifibrotic effects despite of elevated levels of HGF.

The second possibility is the notion that the proliferative state of target cells may influence the effects of HGF on ECM turnover. It had been demonstrated that HGF increases production of ECM in renal cells [50] and induces profibrotic changes in peritoneal cells [51]. Based on the observation that there is a relationship between cell proliferation and production of ECM molecules [52] and that the effects and expression of growth factors may be altered by ECM components [53], Esposito showed that HGF affects quiescent and proliferating renal tubular epithelial (HK-2) cells differently, favoring deposition of ECM in proliferating and matrix degradation in quiescent HK-2 cells [54]. Clinically, antifibrotic therapy often is initiated without control of the underlying disease process and therefore liver cells would be in a proliferative state. On the other hand, in some experimental liver fibrosis reports, the fibrogenic agent is discontinued before or at the initiation of antifibrotic therapy favoring the transition of liver cells from the proliferative to the quiescence state during the spontaneous recovery phase [24], [47]. Our results showed that during the proliferative phase of active fibrogenesis while the hepatotoxic agent is still being administered, overexpression of exogenous HGF combined with the elevated TIMP-1 levels may have accounted for the vigorous stimulation of cell proliferation and ECM production and consequently resulted in less effective remodeling of ECM during this early phase of fibrosis resolution. This is supported by the absence of reduction in collagen content, α-SMA-stained cells, TGF-β1, hydroxyproline concentration and fibrosis score in the HGF-treated fibrotic livers.

A third argument would be the development of antigenic reaction to the transferred human HGF. Kusumoto K et al. showed that treatment of hepatic fibrosis with recombinant human HGF (rh-HGF) was associated with increased urinary albumin excretion [55]. Subsequently, Mizuno et al. showed that rh-HGF, but not rat HGF, elicits glomerular injury and albuminuria in normal rats via an immune complex-dependent mechanism. Furthermore, this immune complex stimulated the production of proteinuric cytokines (including TGF-β) in rat cultured mesangial cells. In contrast, treatment of healthy rats with rat HGF for 4 weeks caused neither mesangial IgG deposition nor elevated anti-HGF IgG in the blood [56]. It is known that rh-HGF is antigenic to rats due to an 8% difference in amino acid sequences [57].

In this study, we demonstrated that ASH1 and EZH2 lysine methyltransferases were markedly induced during progression of liver fibrogenesis. Moreover, we showed for the first time that, regardless of the mechanisms, resolution of CCl_4_-induced liver fibrosis is regulated, at least in part, by repression of the histone lysine modifying enzymes ASH1 and EZH2 methyltransferases. Furthermore, ASH1 and EZH2 methyltransferases were more repressed in livers of mMMP-9-treated than in livers of HGF-treated animals which may have accounted for the more effective resolution of liver fibrosis in the mMMP-9-treated animal group.

ASH1 methyltransferase catalyzes the methylation of K4 on H3 leading to gene activation, while EZH2 is involved in methylation of K 27 on H3 resulting in heterochromatin formation and gene repression [58]. Only recently, very few studies investigated the role of histone lysine methyltransferases ASH1 and EZH2 during the progression of liver fibrosis. Perugorria et al. demonstrated that expression of ASH1 histone methyltransferases is highly up-regulated during the transdifferentiation of both rat and primary human HSCs and that myofibroblasts from human livers of varied pathologies including alcoholic liver disease, nonalcoholic steatohepatitis, and primary biliary cirrhosis also express ASH1. The authors found that ASH1 directly binds to the proximal promoter and 5′ end of α-SMA, collagen I, TIMP-1 and TGF-β1 genes in rat activated HSCs and that ASH1 exerts its positive effect on profibrogenic gene transcription by increasing the level of lysine 4 trimethylation at histone H3 [28]. They further showed that reduction in ASH1 using siRNA led to a significant decrease in target gene transcription, with the exception of α-SMA, which appears to be under other regulatory mechanism. Unfortunately, the absence of selective pharmacologic inhibitors of ASH1 limits in vivo studies addressing their role in enhancing resolution of liver fibrosis [28].

Mann et al. showed that EZH2 mRNA and protein expression was absent in qHSC and was induced in HCS transdifferentiation in vitro and in vivo in response to bile duct ligation and CCl_4_ injury [32]. They documented that EZH2 is a regulator of PPARγ gene transcription by showing that siRNA-mediated knockdown of EZH2 or administration of the EZH2 inhibitor (3-deazaneplanocin A, DZNep) in myofibroblasts resulted in elevated expression of PPARγ transcript. H3K27 methylation was barely detected at the PPARγ gene in qHSCs. In activated HSCs, however, induced expression of EZH2 and H3K27 methylation of the 3′ end of PPARγ cause repression of this gene [32]. PPARγ is a negative regulator of several activation markers such as the expression of α-SMA, type I collagen, and TGF-β1 and other phenotypic characteristics of the myofibroblast [59]. Transcriptional silencing of the PPARγ gene is required for conversion of hepatic stellate cells (HSC) into myofibroblasts [32].

Subsequently Yang et al. confirmed the role of EZH2 in liver fibrosis. They investigated the molecular mechanism underlying the effect of herbal prescription Yang-Gan-Wan in preventing liver fibrosis. They first identified the polyphenolic rosmarinic acid (RA) and baicalin (BC) as the active phytocompounds responsible for the antifibrotic effects. Then, they showed that RA and BC suppressed the expression of EZH2 methyltransferase with consequent reduction of PPARγ H3K27di-methylation which resulted in the formation of euchromatin and increased transcription of PPARγ leading to inhibition of HSC activation and progression of liver fibrosis [60]. Taken together, these experiments and ours confirm the coordinated involvement of active (ASH1 H3K4 methylation) and repressive (EZH2 H3K27 methylation) epigenetic marks in progression and resolution of liver fibrosis. Future single or combined pharmacologic manipulation of these methyltransferases should be evaluated as a novel therapeutic strategy to enhance the resolution of liver fibrosis.

In conclusion, we have demonstrated that mMMP-9 and HGF gene therapy reduce CCl_4_-induced liver fibrosis in rats. Future translational studies should test the effectiveness of human MMP-9 mutants in the resolution of liver fibrosis. We have also showed that ASH1 and EZH2 methyltransferases are elevated during fibrosis progression and are repressed during the early phase of resolution of liver fibrosis. Prospective investigations should explore the therapeutic effect of selective pharmacologic inhibitors of ASH1 and EZH2 in the resolution of liver fibrosis.

## Supporting Information

File S1 **Supporting information.** Table S1, primer sequence and amplicon size. Figure S1, Adenovirus E4 gene detection. Representative gel shows E4 PCR product (714 bp) in the liver at day 7 after second injection of vectors encoding HGF, HGF+ mMMP-9 and mMMP-9. No E4 product was produced in the saline-treated fibrotic group. Figure S2, MT stained livers. Representative photomicrographs of MT-stained liver sections showing A, normal liver with no fibrosis (fibrosis score of 0); B, liver with fibrous expansion of the portal areas (fibrosis score of 1); C, liver with marked septal fibrosis (fibrosis score of 2); D, liver with portal-portal septa, bridging fibrosis but intact architecture (fibrosis score of 3); E, liver with bridging fibrosis with nodules; advanced fibrosis (fibrosis score of 4).
(DOCX)
