# Supplementary material for: Mutant MMP-9 and HGF Gene Transfer Enhance Resolution of CCl4-Induced Liver Fibrosis in Rats: Role of ASH1 and EZH2 Methyltransferases Repression
Source: PLoS One. 2014 Nov 7;9(11):e112384. doi: 10.1371/journal.pone.0112384 (PMC4224431; doi:10.1371/journal.pone.0112384)

File S1. Supporting Information

Supporting Information Figure Legends

**Figure S1.** Adenovirus E4 gene expression. Representative gel shows E4 PCR product (714 bp) in the liver at day 7 after second injection of vectors encoding HGF, HGF + mMMP-9 and mMMP-9. No E4 product was produced in the saline-treated fibrotic group.

**Figure S2. MT stained livers.** Representative photomicrographs of MT-stained liver sections showing A, normal liver with no fibrosis (fibrosis score of 0); B, liver with fibrous expansion of the portal areas (fibrosis score of 1); C, liver with marked septal fibrosis (fibrosis score of 2); D, liver with portal-portal septa, bridging fibrosis but intact architecture (fibrosis score of 3); E, liver with bridging fibrosis with nodules; advanced fibrosis (fibrosis score of 4).

Supporting Information

Table S1. Primer sequence and amplicon size

| **Gene** | **Sequence** | **Size (bp)** |
| --- | --- | --- |
| mMMP-9 | F: 5ʹ-TTT CCC CCT GGC GCC GGC GTT-3ʹ  R: 5ʹ-CTC GTG CGC TGC CAC CAGG-3ʹ | 633 |
| HGF | F: 5ʹ-GCC TGA AAG ATA TCC CGA CA-3ʹ  R: 5ʹ-TTC CAT GTT CTT GTC CCA CA-3ʹ | 523 |
| TIMP-1 | F: 5ʹ-CCT TGC AAA CTG GAG AGT GACA-3ʹ  R: 5ʹ-AGG CAA AGT GAT CGC TCT GGT-3ʹ | 91 |
| Col1α1 | F: 5ʹ-TTC CCT GGA CCT AAG GGT ACT-3ʹ  R: 5ʹ-TTG AGC TCC AGC TTC GCC-3ʹ | 114 |
| α-SMA | F: 5ʹ-ACT GGG ACG ACA TGG AAA AG-3 ʹ  R: 5ʹ-CAT CTC CAG AGT CCA GCA CA-3ʹ | 240 |
| TGFβ1 | F: 5ʹ-TTG CCC TCT ACA ACC AAC ACAA-3ʹ  R: 5ʹ-GCT TGC GAC CCA CGT AGTA-3ʹ | 103 |
| PPARγ | F: 5ʹ-ATT CTG GCC CAC CAA CTT CGG-3ʹ  R: 5ʹ-TGG AAG CCT GAT GCT TTA TCC CCA-3ʹ | 393 |
| E4 | F: 5ʹ-TGT GAC TGA TTG AGC GGTG-3ʹ  R: 5ʹ-CCCATT TAA CAC GCC ATG CA-3ʹ | 714 |
| GAPDH | F: 5ʹ-CAG TGC CAG CCT CGT CTC AT-3ʹ  R: 5ʹ-AGG GGC CAT CCA CAG TCT TC-3ʹ | 595 |
| 18S | F: 5'-TTG ACG GAA GGG CAC CAC CAG-3'  R: 5'-GCA CCA CCA CCC ACG GAA TCG-3' | 131 |

Supplemental Figure S1


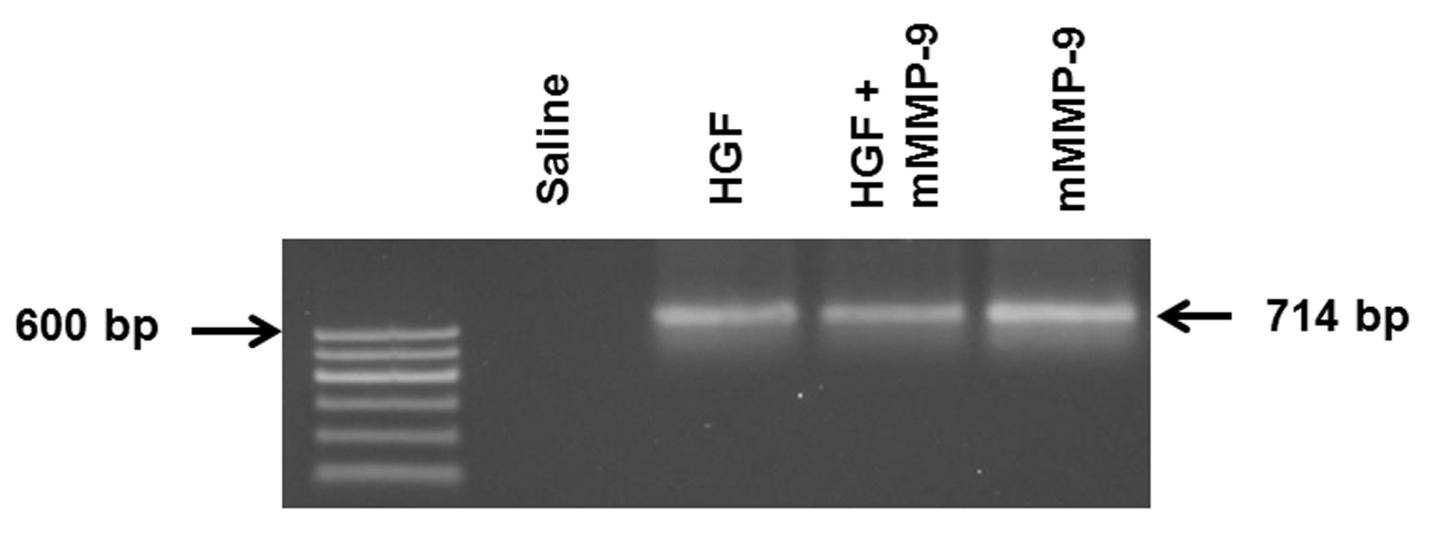


Supplemental Figure S2


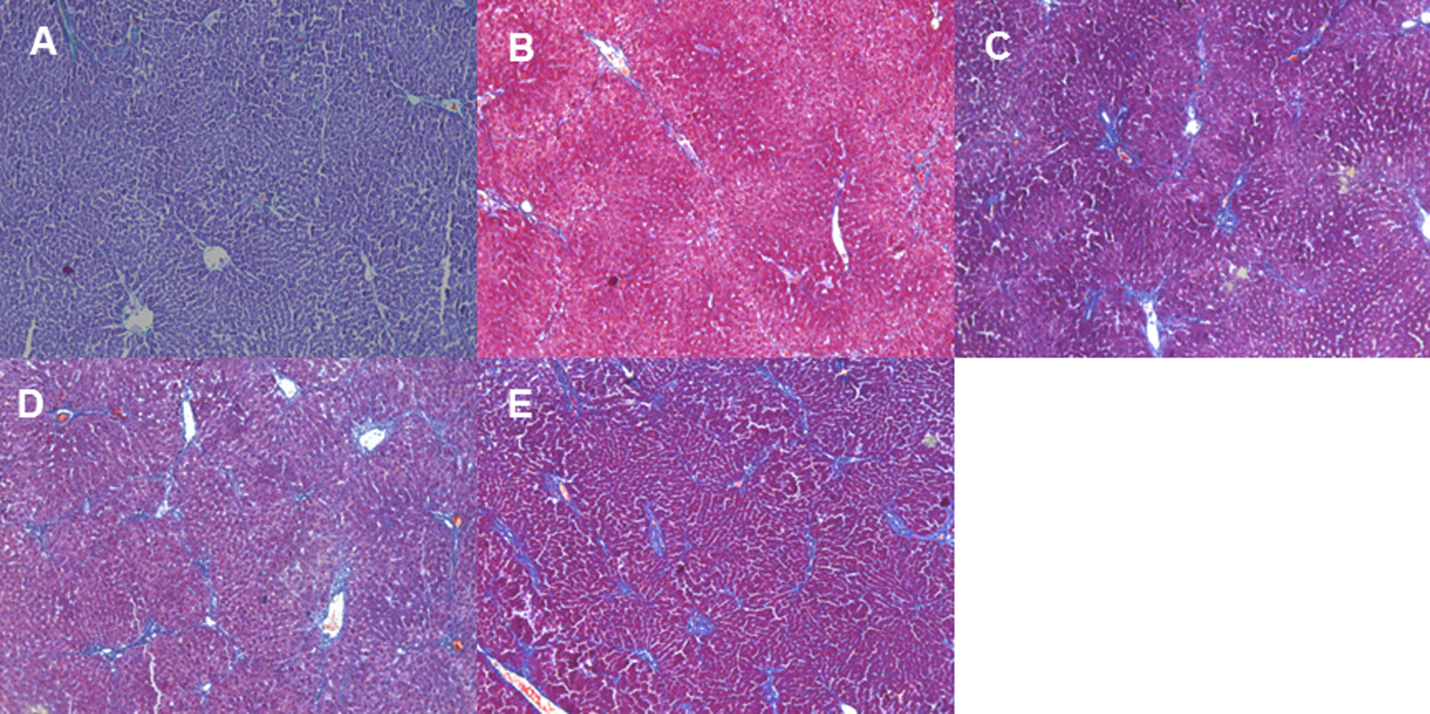

Supplement: File S1 — Supporting information. Table S1, primer sequence and amplicon size. Figure S1, Adenovirus E4 gene detection. Representative gel shows E4 PCR product (714 bp) in the liver at day 7 after second injection of vectors encoding HGF, HGF+ mMMP-9 and mMMP-9. No E4 product was produced in the saline-treated fibrotic group. Figure S2, MT stained livers. Representative photomicrographs of MT-stained liver sections showing A, normal liver with no fibrosis (fibrosis score of 0); B, liver with fibrous expansion of the portal areas (fibrosis score of 1); C, liver with marked septal fibrosis (fibrosis score of 2); D, liver with portal-portal septa, bridging fibrosis but intact architecture (fibrosis score of 3); E, liver with bridging fibrosis with nodules; advanced fibrosis (fibrosis score of 4). (DOCX) [file pone.0112384.s001.docx]
